# Insight into the Epidemiology and Evolutionary History of Novel Goose Astrovirus-Associated Gout in Goslings in Southern China

**DOI:** 10.3390/v14061306

**Published:** 2022-06-15

**Authors:** Xinliang Fu, Zhanpeng Hou, Wenjun Liu, Nan Cao, Yu Liang, Bingxin Li, Danli Jiang, Wanyan Li, Danning Xu, Yunbo Tian, Yunmao Huang

**Affiliations:** 1 College of Animal Science & Technology, Zhongkai University of Agriculture and Engineering, Guangzhou 510225, China; fuxinliang1@163.com (X.F.); houzhanpeng0406@163.com (Z.H.); lwjhero123@126.com (W.L.); caonan870405@126.com (N.C.); 13144174608@163.com (Y.L.); libingxin212@126.com (B.L.); danli0222@163.com (D.J.); lwanyan88@126.com (W.L.); xdanning212@126.com (D.X.); tyunbo@126.com (Y.T.); 2 Guangdong Province Key Laboratory of Waterfowl Healthy Breeding, Guangzhou 510225, China

**Keywords:** GoAstV, gout, molecular epidemiology, evolutionary history

## Abstract

A novel gout disease, characterized by visceral urate deposition with high-mortality, with outbreaks in goslings in China since 2016 was caused by a novel goose astrovirus (GoAstV) and resulted in serious economic loss. However, the epidemiology and variation of the GoAstV in goslings in southern China and its evolutionary history as well as the classification of the GoAstV are unclear. In the present study, systematic molecular epidemiology, and phylogenetic analyses of the GoAstV were conducted to address these issues. Our results showed that the GoAstV is widespread in goslings in southern China, and the genomes of six GoAstV strains were obtained. Two amino acid mutations (Y36H and E456D) were identified in capsid proteins in this study, which is the dominant antigen for the GoAstV. In addition, the GoAstV could be divided into two distinct clades, GoAstV-1 and GoAstV-2, and GoAstV-2 is responsible for gout outbreaks in goslings and could be classified into *Avastrovirus* 3 (AAstV-3), while GoAstV-1 belongs to *Avastrovirus* 1 (AAstV-1). Moreover, the emergence of GoAstV-2 in geese was estimated to have occurred in January 2010, approximately 12 years ago, while GoAstV-1 emerged earlier than GoAstV-2 and was estimated to have emerged in April 1985 based on Bayesian analysis. The mean evolutionary rate for the GoAstV was also calculated to be approximately 1.42 × 10^−3^ nucleotide substitutions per site per year. In conclusion, this study provides insight into the epidemiology of the GoAstV in goslings in southern China and is helpful for understanding the origin and evolutionary history as well as the classification of the GoAstV in geese.

## 1. Introduction

Astroviruses (AstVs) are nonenveloped, positive sense, single-stranded RNA viruses that belong to the Astroviridae family, with capsid shells that are approximately 35 nm in diameter [1]. The astrovirus genome is approximately 6.2 to 7.7 kb and consists of a 5′-untranslated region (UTR); three overlapping open reading frames (ORFs), designated ORF1a, ORF1b, and ORF2; a 3′ UTR; and a poly A tail. The ORF1a encodes a nonstructural polyprotein (serine protease), the ORF1b encodes a polyprotein including RNA-dependent RNA polymerase (RdRp), and the ORF2 encodes the capsid (Cap) protein [2]. Currently, two genera of astrovirus have been recognized, *Mamastrovirus* (MAstV) and *Avastrovirus* (AAstV), which are known to infect mammalian and avian species, respectively [3]. Astrovirus originating from humans and other mammals, including pigs, dogs, sheep, and cattle, belongs to MastV, which typically causes gastroenteritis and, in rare cases, causes neurological syndromes and encephalitis [4]. Astrovirus isolated from avian species, such as turkeys, chickens, ducks, and other birds, is classified into the genus AAstV, which causes diverse pathologies, including enteritis, hepatitis, and nephritis [5]. Three species, *Avastrovirus* 1 (AAstV-1), *Avastrovirus* 2 (AAstV-2), and *Avastrovirus* 3 (AAstV-3), within AAstV have been officially recognized by the International Committee for Taxonomy of Viruses (ICTV). AAstV-1 includes turkey astrovirus 1 (TAstV-1), AAstV-2 includes avian nephritis virus 1 (ANV1) and avian nephritis virus 2 (ANV2), and AAstV-3 includes turkey astrovirus 2 (TAstV-2) and duck astrovirus 1 (DAstV-1) [6]. In addition, there are numerous yet-to-be classified AAstVs, including turkey astrovirus 3 (TAstV-3), chicken astrovirus (CAstV), DAstV-2, DAstV-3, DAstV-4, and diverse viruses isolated from wild birds [3,7].

Since 2016, a novel gout disease, characterized by visceral urate deposition, hemorrhage, and swelling of the kidneys, has outbreaks in goslings in China, causing 10% to 50% mortality in infected goslings, which has caused serious economic losses in the goose industry of China [8,9]. Subsequently, a novel goose astrovirus (GoAstV) was identified and has been confirmed by several research groups as the causative agent for gout outbreaks in goslings [10,11,12]. In addition to goslings, ducks have also been reported to be infected by this novel astrovirus and show similar clinical symptoms to infected goslings, which indicates the possibility of cross-species transmission of the novel GoAstV between domestic waterfowl [13,14]. Moreover, the GoAstV has also been reported to be vertically transmitted from breeding geese to goslings, which thus poses a serious threat to the goose industry.

To address the epidemiology and evolutionary history of the GoAstV in goslings in southern China, clinical samples were collected from farms experiencing gout disease in Guangdong and Fujian Provinces for this study. Six nearly full-length GoAstV genomes were obtained from these samples, and systematic molecular characteristics and evolutionary analysis for the GoAstV were performed. In addition, Bayesian analysis was also conducted to address the origin and evolutionary history of the GoAstV. Our results indicate that the GoAstV is widespread in geese in southern China and that there are two different clades of the GoAstV (e.g., GoAstV-1 and GoAstV-2) circulating in geese. Moreover, GoAstV-2, which causes gout in goslings, emerged in approximately 2010, with 1.42 × 10^−3^ nucleotide substitutions per site per year. This study is helpful for understanding the epidemiology of the GoAstV in goslings in southern China and for gaining insights into the evolutionary history and classification of the GoAstV.

## 2. Materials and Methods

### 2.1. Clinical Signs Description and Sample Collection

From April 2019 to December 2019, eleven goose farms experienced gout outbreaks in goslings in Guangdong and Fujian Provinces, China. The geese in these farms presented with clinical signs of gout, including urate deposits in the joints and ureter and on the surfaces of kidneys and livers, as well as swelling of the kidneys. Tissue samples from 83 diseased goslings (including liver, spleen, and kidney) were collected from these farms for virus detection and histopathologic examinations.

### 2.2. Sample Processing, Virus Detection and Genome Sequencing

The kidney, spleen and liver samples were mixed and homogenized with sterile phosphate buffered saline (PBS, pH 7.4) to a 20% suspension (*w*/*v*) using tissue homogenizer and were centrifuged at 10,000 rpm/min at 4 °C for 10 min. The supernatants were collected for RNA extraction and further usage. Virus RNA was extracted from the supernatants using a viral RNA kit (Omega, Norcross, GA, USA), and cDNA was synthesized using the PrimeScript^TM^ 1st Strand cDNA Synthesis Kit (TAKARA, Dalian, China) with random primers by following the manufacturer’s instructions. The GoAstV detection was tested by RT–PCR with specific primers, and six pairs of primers (Table 1) were designed based on the GoAstV reference strain, AAstV/Goose/CHN/2017/SD01 (MF772821), to amplify the GoAstV genome. In addition, Newcastle disease virus (NDV), influenza A virus (IAV), Tembusu virus (TMUV) and goose parvovirus (GPV) were also detected in these samples, as previously described [12,15]. The PCR products were analyzed by electrophoresis on 1.0% agarose gels and purified using the Gel Extraction Kit (Omega, Norcross, GA, USA). Purified DNA was then ligated into the pMD-18T cloning vector (TAKARA, Dalian, China) and transformed into DH5-α competent cells. Positive recombinant plasmids were sequenced using the Applied Biosystems ABI3730 system (BGI, Beijing, China). Sequences of the genomic fragments were assembled using the DNAStar program to produce the genome sequence of the GoAstV and were used for further analysis.

### 2.3. GoAstV Genome, Molecular Characteristics, Recombination and Phylogenetic Analysis

The complete genome sequence of the GoAstV and the deduced amino acid sequences of ORF1a, ORF1b and ORF2 were compared to those of other reported astroviruses. Multiple sequence alignments were performed using MAFFT software (https://mafft.cbrc.jp/alignment/software/, accessed on 20 July 2021), and phylogenetic tree was constructed based on the nucleotide sequences of the GoAstV genome and the Cap and RdRp genes by using the neighbor-joining method with 1000 bootstrap replicates in MEGA5.0 software [16]. The potential recombination between the GoAstV and other AstVs was tested by bootscan analysis using Simplot 3.5.1 software.

### 2.4. Evolutionary Dynamics and the Evolutionary History of GoAstV

The Bayesian Markov chain Monte Carlo (MCMC) method was used to substantiate the evolutionary dynamics and the divergence time of the GoAstV with other AstVs based on the RdRp gene in BEAST 1.8.3, as previously described [17]. Specifically, the general time-reversible substitution model with a proportion of invariant sites and gamma distributed rate heterogeneity (GTR + I + Γ) was used with Bayesian inference (BI) methods. A relaxed molecular clock with an uncorrelated log-normal rate distribution was used to model the rate variations among branches with a Bayesian skyline coalescent model [18]. The MCMC algorithm was run for a 1 × 10^7^ step chain and sampled every 1000 states, and the first 10% of the chain was removed as burn-in. The maximum clade credibility (MCC) tree was inferred using the Tree Annotator program included in the BEAST package, and the MCC tree was viewed using FigTree software (http://tree.bio.ed.ac.uk/software/figtree/, accessed on 8 August 2021). The mean time of the most recent common ancestor (TMRCA) and the highest posterior density (HPD) regions at 95%, as well as the nucleotide substitution rate (per site per year) for astrovirus, were calculated in Tracer 1.6 (http://beast.bio.ed.ac.uk/software/tracer/, accessed on 8 August 2021) as previously described [19].

## 3. Results

### 3.1. Clinical Signs and Virus Detection

**Figure 1:**
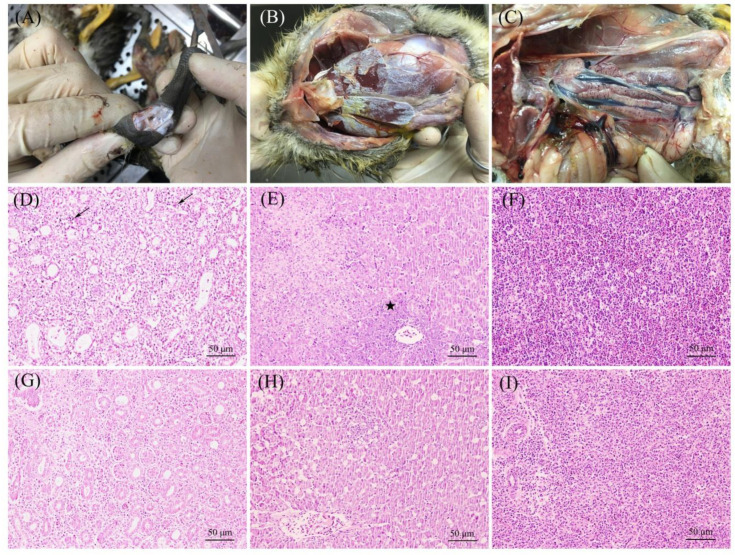
Postmortem lesions and histopathologic changes of goslings presented with gout clinical signs. (**A**–**C**) Urate deposits in the joints and ureter, on the surface of livers, and swelling of the kidneys. (**D**) Swelling of glomeruli and necrosis of renal tubular epithelial cells, as well as inflammatory cell infiltration (arrow) in kidneys (H&E). (**E**) Necrosis of hepatocytes and inflammatory cell infiltration (star) in the liver (H&E). (**F**) Splenic lymphocyte necrosis and hyperplasia (H&E). (**G**–**I**) Normal kidney, liver and spleen HE sections of an uninfected gosling (H&E).

All diseased goslings presented with typical clinical gout signs as previously described [9,10], including depression, joint swelling, and inability to stand, and the mortality rates ranged from 15% to 37% at the eleven affected goose farms. Postmortem examinations revealed urate deposits in the joints and ureter, on the surface of kidneys and livers, as well as swelling of the kidneys (Figure 1A–C). Histopathologic changes in the kidney showed swelling of the glomeruli, necrosis, and abscission of renal tubular epithelial cells, as well as inflammatory cell infiltration in the diseased goslings (Figure 1D). The necrosis of hepatocytes and inflammatory cell infiltration were obvious in the liver (Figure 1E), and the spleen showed splenic lymphocyte necrosis and hyperplasia (Figure 1F). The GoAstV was detected at all affected farms (11/11), and the positivity rate for the samples collected from diseased goslings was approximately 90.4% (75/83), which means that the GoAstV is widespread in goslings in southern China. However, all collected samples were negative for NDV, IAV, TMUV and GPV in this study.

### 3.2. Genome Sequence and Molecular Characteristics Analysis

Six nearly full-length genome sequences (7033 bp) of the GoAstV were obtained in this study, including GoAstV/Guangdong/JYF6/2019 (JYF6), GoAstV/Guangdong/QY1/2019 (QY1), GoAstV/Guangdong/NH2/2019 (NH2), GoAstV/Guangdong/DBN1/2019 (DBN1), GoAstV/Guangdong/DBN30/2019 (DBN30), and GoAstV/Fujian/LWY5/2019 (LWY5), and the GenBank numbers for these six strains range from ON049467 to ON049473. The nucleotides sequences of the six GoAstV strains are 99.2% to 99.6% identical to each other in the full-length genome and are 97.1% to 99.7% identical to other reported GoAstV strains related to gout disease in goslings. A comparison of the GoAstV identified in this study (GoAstV/Guangdong/QY1/2019 as the representative) with other known avian AstVs was conducted. The results showed that the GoAstV was most identical to TAstV-2 based on the complete genome sequence (approximately 63.0%), as well as the amino acid sequences of the ORF1a (59.1%), RdRp (68.3%) and Cap (56.3%) proteins (Table 2), followed by DAstV-2 (SL1). Interestingly, GoAstV-QY1 identified in this study only had 58% identity to a previously reported GoAstV strain (e.g., GoAstV-FLX, named GoAstV-1 later), which is related to enteritis in geese [20].

To determine the variations in the GoAstV in different strains, amino acid comparisons of the Cap and RdRp proteins of the GoAstV identified in this study with reference strains were performed. Two amino acid mutations (K357R and N454S) were found in GoAstV-QY1 and GoAstV-JYF6 in the RdRP protein. In addition, all six identified GoAstV strains contain the Y36H mutation, and four strains (e.g., QY1, JYF6, LWY5 and DBN30) have the E456D mutation in the Cap protein. Notably, the E456D mutation was located in the spike part of the Cap protein, which is the dominant antigen for AstVs. Thus, the influence and significance of these mutations need to be further investigated.

### 3.3. Phylogenetic Analysis and Classification of GoAstV

**Figure 2:**
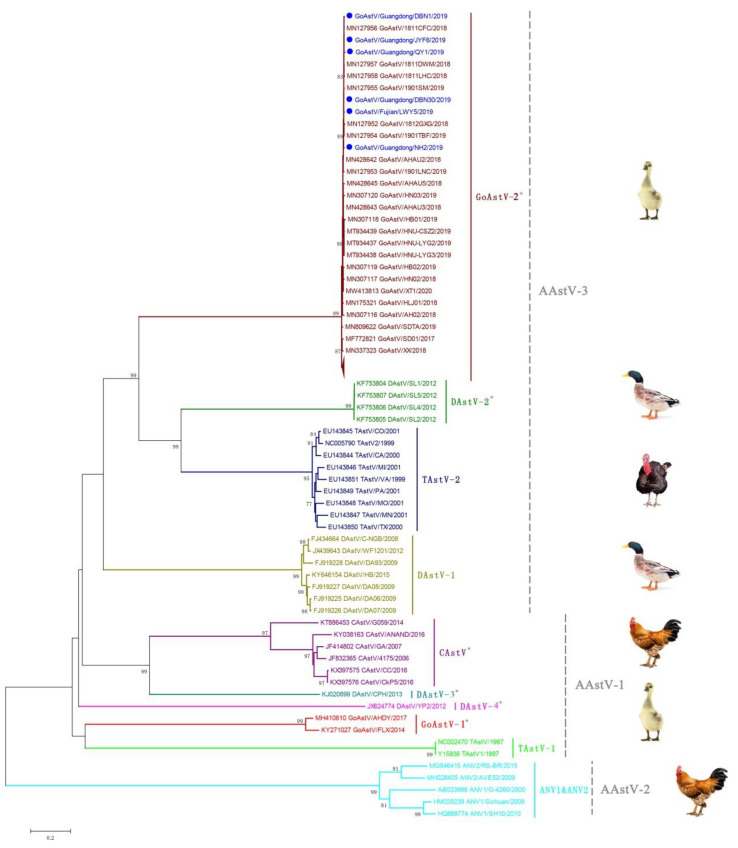
Phylogenetic analysis of the GoAstV with other astroviruses constructed based on the complete genome. The tree was constructed with the neighbor-joining method with 1000 bootstrap replicates in MEGA 5.0 after multiple sequence alignments by MAFFT. The strains shown in blue with solid blue circles were identified in this study, and proposed species yet to be recognized are designated with asterisks.

**Figure 3:**
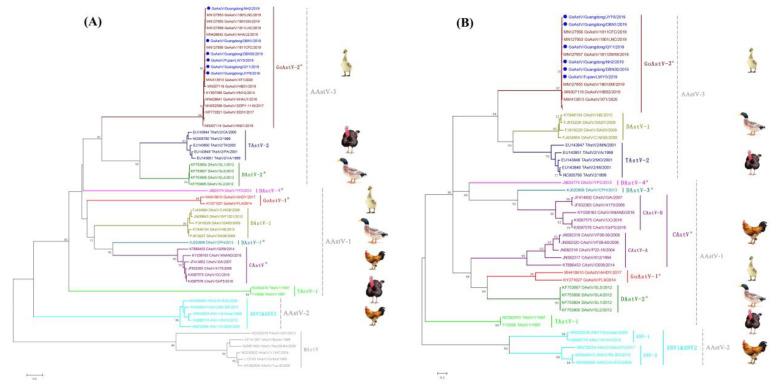
Phylogenetic analysis of the GoAstV with other astroviruses constructed based on the RdRp and Cap genes. (**A**) Phylogenetic tree of astroviruses constructed by using the RdRp gene (1551 bp) and (**B**) phylogenetic tree of astroviruses constructed by using the Cap gene (2115 bp). The strains shown in blue with solid blue circles were identified in this study, and proposed species yet to be recognized are designated with asterisks.

To further understand the evolutionary relationship of the GoAstVs with other AAstVs, a systematic phylogenetic analysis was conducted based on the nucleotide sequences of whole genomes, RdRp genes and Cap genes. The results showed that all the GoAstVs can be divided into two distant clades, named GoAstV-1 and GoAstV-2, and the six GoAstV strains identified in this study belongs to clade GoAstV-2 (Figure 2 and Figure 3). GoAstV-2 has a closer phylogenetic relationship with TAstV-2 and DAstV-2 based on the phylogenetic analyses of whole genomes and RdRp genes (Figure 2 and Figure 3A), while GoAstV-2 is close to TAstV-2 and DAstV-1 based on phylogenetic analyses of the Cap genes (Figure 3B), which coincides with the results of nucleotide identities analysis in Table 2.

Based on the phylogenetic analysis of the full-length Cap gene with officially classified strains, the GoAstV-2 could be classified into AAstV-3, while the GoAstV-1 belongs to AAstV-1 in the present study (Figure 3B). Recombination detection was also conducted, but no recombination single was detected (data not shown). The mean amino acid genetic distance (p-distance) of the Cap protein is another standard for AstV species or genotype species classification [21]. The p-distances of the Cap protein between GoAstV-2 and other AAstV species were calculated, and the genetic distance between GoAstV-2 and GoAstV-1 was 0.609 ± 0.021. In addition, the genetic distances among GoAstV-2 and other AAstVs ranged from 0.399 ± 0.021 to 0.709 ± 0.020 (Table 3). These results indicate that there are two independent astrovirus species circulating in geese.

### 3.4. Divergence Time and Evolutionary History of GoAstV-2

**Figure 4:**
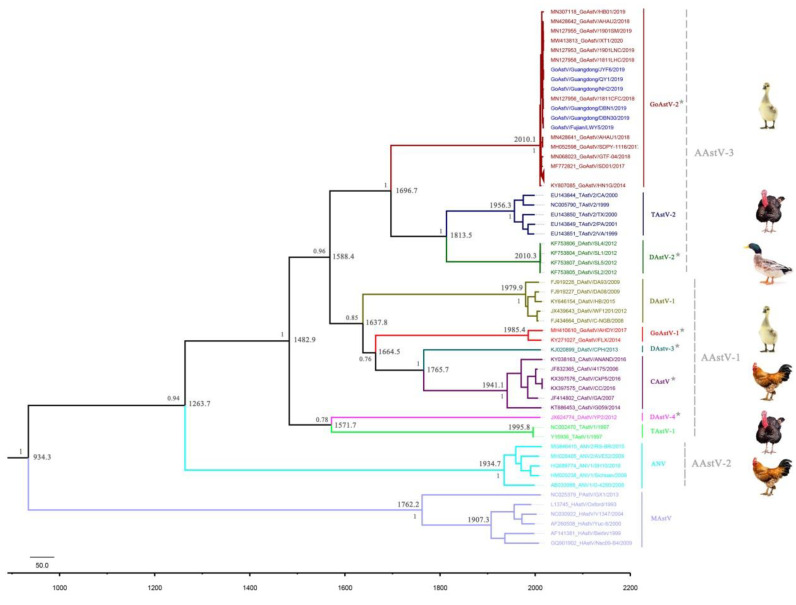
Bayesian maximum clade credibility (MCC) phylogenetic tree constructed in BEAST 1.8.3 using the Markov chain Monte Carlo (MCMC) method based on the RdRp gene. The mean TMRCA was estimated for each key node, and the high posterior probability values are shown for each key node and provide an assessment of the degree of support for the node on the tree. The strains shown in blue color were identified in this study, and proposed species yet to be recognized are designated with asterisks.

The MCC tree constructed from the Bayesian analysis has a topology that is similar to the phylogenetic tree constructed using the neighbor-joining method based on the RdRp gene, with high posterior probability values supporting each key node, and the mean TMRCA was estimated for each key node (Figure 4). The mean TMRCA for all gout-associated strains in GoAstV-2 was estimated as 2010.1 (95% HPD, 2007.2 to 2012.1), which means that GoAstV-2 emerged approximately 12 years ago. The divergence time between GoAstV-2 and TAstV-2/DAstV-2 was estimated at 1696.7 (95% HPD, 1524.1 to 1802.5), and the mean TMRCA for GoAstV-1 was estimated at 1985.4 (95% HPD, 1962.7 to 2000.2), which means that GoAstV-1 emerged earlier than GoAstV-2. The TMRCA for all AAstVs, including AAstV-1, AAstV-2, and AAstV-3, was estimated at 1263.7 (95% HPD, 857.9 to 1534.8), indicating that the AAstVs have circulated in avian species and evolved for nearly 750 years. In addition, the TMRCA for the AAstVs and the MAstVs was estimated to be approximately 934.3 (95% HPD, 328.2 to 1385.7) in this study. The mean evolutionary rate of the RdRp gene for the AAstVs was also calculated by Bayesian analysis, and the mean variation rate was approximately 1.42 × 10^−3^ (95% HPD, 0.93 × 10^−3^ to 1.99 × 10^−3^) nucleotide substitutions per site per year.

## 4. Discussion

*Avastrovirus* infections have been reported in multiple avian species, including turkeys, chickens, geese, ducks, and many kinds of wild aquatic birds and cause diverse pathologies, such as enteritis, hepatitis and nephritis [5]. However, a novel GoAstV that mainly causes gout in gosling has spread with outbreaks in China since 2016, which has led to enormous economic losses to the goose industry [8,9]. Moreover, the GoAstV has been reported to be vertically transmitted from breeding geese to goslings, which poses a potential challenge to the prevention of the GoAstV [22]. Niu et al. reported that the infection rate of the GoAstV in clinical samples collected from six provinces in North China was 81.5% [9]. In addition, the epidemiology of the GoAstV in goslings have been conducted by several study groups in different regions, and the results showed that the GoAstV infections in goslings are widespread [23,24,25]. In the present study, the epidemiology of the GoAstV in goslings in southern China was examined, and the results showed that the GoAstV is also prevalent in goslings in southern China. The GoAstV can be detected in multiple tissues in infected goslings, including the kidney, liver, lung, spleen, and brain, which indicates that it has a wide tissue tropism [11]. The level of kidney damage was severe, and histopathologic changes were typical in this study and were also observed in previous research, which included swelling of glomeruli, necrosis, and abscission of renal tubular epithelial cells. Moreover, the virus titre in the kidney is higher than that in other tissues after the GoAstV infection [10], and these results reveal that the kidney may be the major target tissue for the GoAstV infections.

The full-length Cap gene was recommended as the genetic criteria for use in the AstV species classification by ICTV, and the Cap protein is the structural protein for AstV and is responsible for antibody production with AstV infection [4,6]. Several amino acid mutations in the Cap protein have been identified in this study, including Y36H and E456D. In addition, other amino acid mutations in Cap protein have also been reported previously, such as Q229P and A614T [23,24], and whether these mutations influence the antigenicity of the GoAstV needs further investigation. Two distant clades of the GoAstV have been identified, and the homology of the whole genome and Cap protein between GoAstV-1 and GoAstV-2 is only 58.0% and 42.5%, respectively. In addition, the genetic distance between GoAstV-1 and GoAstV-2 was 0.609 ± 0.021 (Table 3), which is comparable to the genetic distance (0.576–0.741) between different species of AAstV [6], this means that there are two different astrovirus species circulating in geese. Interestingly, all the GoAstV strains in the GoAstV-2 clade have been associated with gout outbreaks in geese since 2016, while the strains in the GoAstV-1 clade were reported in geese with enteritis as described previously [20]. Moreover, the GoAstV-2 could be classified into AAstV-3 with TAstV-2 and DAstV-2, while GoAstV-1 may be classified into AAstV-1 with TAstV-1, CAstV, DAstV-3 and DAstV-4 based on the phylogenetic relationship of the Cap gene (Figure 3B), and these results will be helpful for the classification of the GoAstV.

Because the RdRp gene is the most conserved gene among all AstVs [26], the RdRp gene was used in the present study for Bayesian analysis to address the divergence time and evolutionary history of the GoAstV-2. As the results shown, the mean TMRCA for HAstV genotype 1 (HAstV/V1347/2004), genotype 3 (HAstV/Berlin/1999), and genotype 6 (HAstV/Nsc09-B4/2009) was estimated to be approximately 1907.3 (95% HPD, 1896.9 to 1967) in the present study, which is highly consistent with a previous study [27] and indicate that the Bayesian analysis in this study is unbiased. The TMRCA for GoAstV-2 was estimated at 2010, which indicated that GoAstV-2 has circulated in geese for approximately 12 years, and this result is coincide with a confirms previous research [28]. However, the emergence time for GoAstV-1 was estimated at 1985.4 in this study, which differs from those reported previously [28]; this may result from using different gene fragment and different numbers of reference strains. Computing the mutation rate is also helpful to address the evolutionary history of the GoAstV, and the variation rates of the RdRp gene for the AAstVs were estimated to be approximately 1.42 × 10^−3^ nucleotide substitutions per site per year in the present study, which is lower than the variation rate of HAstVs, which was 3.7 × 10^−3^ nucleotide substitutions per site per year [27]. The results found in the present study will be helpful for understanding the evolutionary history of the GoAstV.

Insight into the pathogenic mechanism of the GoAstV is critical to reveal the mechanism of gout induced by the GoAstV, and several studies have been conducted to address this objective. Wu et al. reported that the GoAstV infections could cause lesions on the liver and kidney, which result in the increased production of uric acid in the liver and impede the excretion of uric acid in the kidney, which contribute to hyperuricemia and gout formation [29]. Host innate immune responses of geese infected with the GoAstV have also been reported. Pattern recognition receptors (PRRs), including RIG-I/MDA5 and TLR3, are involved in the host immune response to the GoAstV, and the expressions of IFN types I (e.g., IFN-α and IFN-β), inflammatory cytokines (e.g., IL-8, IL-10, and TNF-α) and antiviral proteins (e.g., MX1, OASL, IFITM3, PKR) are upregulated in the kidney and spleen induced by the GoAstV infections, which have been reported previously [30,31,32]. However, the molecular mechanism of the severe lesions in the kidney and liver that are caused by the GoAstV needs to be further investigated, which is critical for revealing the mechanism of gout induced by the GoAstV.

In conclusion, systematic molecular epidemiology and phylogenetic analyses were conducted in the present study, and we gained insight into the epidemiology, molecular features, and evolutionary history as well as the classification of the GoAstV, which is helpful for understanding the classification and origin of the GoAstV.

## Figures and Tables

**Table 1:** Primers used for RT–PCR detection and genome amplification of the GoAstV in this study designed by AAstV/Goose/CHN/2017/SD01 (MF772821).

Primer Names	Primer Sequences (5′-3′)	Product Length	Purpose
GoAstV-D: F	TATGCTCCTACTAAATGGGAC	540 bp	virus detection
GoAstV-D: R	ACCAATGAGCCTAGATACTCG		
GoAstV: F1	CCCAAAACCCCTTTTCCTCACT	1380 bp	genome amplification
GoAstV: R1	TAATTGTCAAGGCTGTAGACCAC		
GoAstV: F2	GGTTGAGGTTTCTTATGCGGTA	1286 bp	
GoAstV: R2	CGCCCCTTTCTTCCATTGCT		
GoAstV: F3	AAGAAGGTGCGGAAGAGTGG	1250 bp	
GoAstV: R3	GGGGCCTATTTTGCACAGT		
GoAstV: F4	TGAACAATGTGGCTGGGGTGA	1374 bp	
GoAstV: R4	TATTTATGCTTCCGGTCGAGT		
GoAstV: F5	CTGGACCACCCGTTAATGACA	1198 bp	
GoAstV: R5	CTTGACCTGGATTCTGCCTGT		
GoAstV: F6	GGCTGCAAATACAGTTAAGCTTG	1102 bp	
GoAstV: R6	CACGCGATTTGTGTGGGTGAC		

**Table 2:** Comparison of the GoAstV with other known AstVs based on the genome nucleotide and amino acid sequences of the ORF1a, RdRp and Cap genes.

Astrovirus (GenBank Accession No.)	Genome Size	G + C Content (%)	Percent Identity (%) to GoAstV/Guangdong/QY1/2019			
			Genome (nt)	ORF1a (aa)	RdRp (aa)	Cap (aa)
GoAstV/Guangdong/QY1/2019	7033 bp	43.4	NA ^a^	NA	NA	NA
TAstV-2/CO/2001 (EU143845)	7343 bp	43.5	63.0	59.1	68.3	56.3
DAstv-2/SL1/2012 (KF753804)	7319 bp	42.0	60.7	58.5	68.0	40.1
DAstV-1/HB/2015 (KY646154)	7754 bp	41.8	60.0	48.5	63.9	56.2
DAstV-3/CPH/2013 (KJ020899)	7463 bp	43.0	58.9	50.0	65.4	37.5
GoAstV-1/FLX/2014 (KY271027)	7299 bp	41.6	58.0	47.4	61.0	42.5
DAstV-4/YP2/2012 (JX624774)	7287 bp	43.2	56.9	42.3	61.3	34.3
CAstV-A/G059/2014 (KT886453)	7382 bp	42.5	58.2	49.2	65.0	38.9
CAstV-B/4175/2006 (JF832365)	7008 bp	42.9	58.0	49.1	57.9	32.4
TAstV-1/1997 (NC002470)	7003 bp	43.5	54.6	40.4	56.8	41.3
ANV/SH10/2019 (HQ889774)	6928 bp	44.8	52.2	27.0	53.0	28.0
HAstV/Berlin/1999 (AF141381)	6815 bp	44.1	49.0	17.7	38.6	18.7

Note: ^a^ NA, data not available for analysis. GoAstV, goose astrovirus; TAstV, turkey astrovirus; DAstV, duck astrovirus; CAstV, chicken astrovirus; ANV, avian nephritis virus; and HAstV, human astrovirus.

**Table 3:** The genetic distances (p-distance) between different AAstV species based on the complete amino acid sequences of the Cap protein.

AAstV Species	Average p-Distance between Different AAstV Species (Mean ± SE)									
	GoAstV-1 *	GoAstV-2 *	TAstV-1	TAstV-2	DAstV-1	DAstV-2 *	DAstV-3 *	DAstV-4 *	CAstV *	ANV
GoAstV-2 *	0.609 ± 0.021									
TAstV-1	0.565 ± 0.20	0.594 ± 0.022								
TAstV-2	0.595 ± 0.021	0.403 ± 0.021	0.607 ± 0.020							
DAstV-1	0.595 ± 0.022	0.399 ± 0.021	0.615 ± 0.020	0.261 ± 0.018						
DAstv-2 *	0.424 ± 0.021	0.592 ± 0.021	0.590 ± 0.020	0.588 ± 0.021	0.597 ± 0.021					
DAstV-3 *	0.611 ± 0.021	0.624 ± 0.020	0.624 ± 0.022	0.597 ± 0.021	0.580 ± 0.021	0.595 ± 0.021				
DAstV-4 *	0.605 ± 0.021	0.655 ± 0.021	0.632 ± 0.019	0.624 ± 0.022	0.6260 ± 022	0.595 ± 0.021	0.517 ± 0.021			
CAstV	0.494 ± 0.021	0.603 ± 0.021	0.592 ± 0.021	0.565 ± 0.020	0.582 ± 0.020	0.500 ± 0.021	0.592 ± 0.021	0.588 ± 0.021		
ANV	0.695 ± 0.019	0.709 ± 0.020	0.588 ± 0.020	0.719 ± 0.020	0.721 ± 0.020	0.708 ± 0.020	0.723 ± 0.019	0.712 ± 0.020	0.702 ± 0.020	
HAstV	0.761 ± 0.018	0.823 ± 0.016	0.796 ± 0.017	0.786 ± 0.017	0.784 ± 0.018	0.788 ± 0.017	0.786 ± 0.017	0.773 ± 0.017	0.777 ± 0.017	0.755 ± 0.016

Note: AAstV strains used in this analysis, GoAstV-1: GoAstV-1/FLX/2014 (KY271027), TAstV-1: TAstV-1/1997 (NC002470), TAstV-2: TAstV-2/CO/2001 (EU143845), DAstV-1: DAstV-1/HB/2015 (KY646154), DAstV-2: DAstV-2/SL1/2012 (KF753804), DAstV-3: DAstV-3/CPH/2013 (KJ020899), DAstV-4: DAstV-4/YP2/2012 (JX624774), CAstV: CAstV-A/G059/2014 (KT886453), ANV: ANV/SH10/2010 (HQ889774), and HastV: HAstV/Berlin/1999 (AF141381). Proposed species yet to be recognized are designated with asterisks.

## Data Availability

Not applicable.
